# Enhancing Emergency Department Management: A Data-Driven Approach to Detect and Predict Surge Persistence

**DOI:** 10.3390/healthcare12171751

**Published:** 2024-09-02

**Authors:** Kang Heng Lim, Francis Ngoc Hoang Long Nguyen, Ronald Wen Li Cheong, Xaver Ghim Yong Tan, Yogeswary Pasupathy, Ser Chye Toh, Marcus Eng Hock Ong, Sean Shao Wei Lam

**Affiliations:** 1 Health Services Research Centre, Singapore Health Services Pte Ltd., Singapore 169856, Singapore; limkangheng@u.nus.edu (K.H.L.); nguyen.ngoc.hoang.long@singhealth.com.sg (F.N.H.L.N.); ronald.cheong.w.l@singhealth.com.sg (R.W.L.C.); s10203489@connect.np.edu.sg (X.G.Y.T.); marcus.ong@duke-nus.edu.sg (M.E.H.O.); 2 NUS Business Analytics Centre, NUS Business School, National University of Singapore, Singapore 119245, Singapore; 3 Ngee Ann Polytechnic, Singapore 599489, Singapore; toh_ser_chye@np.edu.sg; 4 Department of Emergency Medicine, Singapore General Hospital, Singapore 169608, Singapore; yogeswary.pasupathi@sgh.com.sg; 5 Health Services and Systems Research, Duke-NUS Medical School, National University of Singapore, Singapore 169857, Singapore; 6 Lee Kong Chian School of Business, Singapore Management University, Singapore 178899, Singapore

**Keywords:** time series, SARIMAX, EWMA, control charts, machine learning, emergency department overcrowding, drift detection

## Abstract

The prediction of patient attendance in emergency departments (ED) is crucial for effective healthcare planning and resource allocation. This paper proposes an early warning system that can detect emerging trends in ED attendance, offering timely alerts for proactive operational planning. Over 13 years of historical ED attendance data (from January 2010 till December 2022) with 1,700,887 data points were used to develop and validate: (1) a Seasonal Autoregressive Integrated Moving Average with eXogenous factors (SARIMAX) forecasting model; (2) an Exponentially Weighted Moving Average (EWMA) surge prediction model, and (3) a trend persistence prediction model. Drift detection was achieved with the EWMA control chart, and the slopes of a kernel-regressed ED attendance curve were used to train various machine learning (ML) models to predict trend persistence. The EWMA control chart effectively detected significant COVID-19 events in Singapore. The surge prediction model generated preemptive signals on changes in the trends of ED attendance over the COVID-19 pandemic period from January 2020 until December 2022. The persistence of novel trends was further estimated using the trend persistence model, with a mean absolute error of 7.54 (95% CI: 6.77–8.79) days. This study advanced emergency healthcare management by introducing a proactive surge detection framework, which is vital for bolstering the preparedness and agility of emergency departments amid unforeseen health crises.

## 1. Introduction

The efficient management of emergency departments (EDs) is critical to providing timely and effective healthcare services to needy patients. EDs often face numerous challenges due to increasing patient volumes, inpatient bed shortages, and unpredictable patient arrivals at varying severity levels [1,2,3]. When poorly managed, lengthy patient waiting times can lead to patients leaving the ED without being treated, foster violence against healthcare staff, and result in increased morbidity and mortality [4,5]. ED overcrowding similarly compromises the quality of treatment and prognosis by medical staff, decreasing physician job satisfaction and reducing patient safety [6,7].

To address the challenges of ED overcrowding, healthcare professionals have turned to making reliable ED attendance predictions in order to optimize resource allocation. Traditional statistical methods such as moving averages, regression analysis, and time series analysis are standard implementations for ED predictions [8,9,10,11,12], with errors ranging between 4.2% and 14.4% for daily attendance predictions [13]. There has been comprehensive coverage of static model building, validation, and testing in the context of ED attendance forecasting [8,9,10,11,12,13,14,15,16,17,18,19]. However, research on model deployment for continuous training and testing is relatively scarce [8,9,10,11,12,13,14,15,16,17,18,19]. Previous research has proposed change point detection methods and control charts to detect outliers and changes in trends of stochastic processes [20]. However, there have been limited methods to address situations with high uncertainty [21,22,23,24,25].

In times of uncertainty, the ability to pre-emptively detect shifts in ED trends is useful for guiding effective policy response strategies. These shifts are notably exemplified by the challenges posed by the COVID-19 pandemic [26,27] and related changes in nationwide healthcare policies [28,29,30]. These interconnected occurrences underscore the need to continuously monitor trends in ED attendance so that stakeholders can appropriately respond to emergent situations and ensure the continued provision of high levels of care.

We aimed to forecast ED attendances using a Seasonal Autoregressive Integrated Moving Average with eXogenous factors (SARIMAX) model that captures calendar fixed effects [13,14,15]. In a comparative analysis of statistical models (e.g., Holt–Winters, ARIMA, SARIMAX) and machine learning models (e.g., LSTM, Gradient Boosting, Random Forest) within the context of ED attendance prediction, the SARIMAX model was chosen due to its offering comparable performances along with advantages in model interpretability and ease of implementation [8,9,10,11,12,13,16]. We propose an Early Warning System for ED attendance predictions (EWS-ED), which allows for continuous monitoring of forecasts for changes to the underlying stochastic processes that drive ED attendance. EWS-ED includes a predictive model for predicting the persistence of these changes. An Exponentially Weighted Moving Average (EWMA) control chart [30,31] was used to first detect anomalies in the quality of forecasts based on the SARIMAX model. A machine learning (ML) model was then trained and validated to predict the persistence of the anomalous conditions. This can enable pre-emptive detection of anomalous trends and allow analysts to decide whether retraining of the model is required.

## 2. Materials and Methods

The study hospital (SH) is one of the largest comprehensive public hospitals in Singapore, comprising more than 30 clinical disciplines and approximately 1900 inpatient beds in 2022. In the same year, the SH saw more than 100,000 ED attendances annually. Daily ED attendance data between 1 January 2010 and 31 December 2022 were extracted from the ED administrative database of the SH. Other data collected for the study included exogenous factors that may affect ED attendance (Table 1) [9,10,11]. Meteorological factors such as ambient temperature, air quality, and relative humidity were omitted, as Singapore is a tropical country with low variability in weather conditions [10].

**Figure 1:**
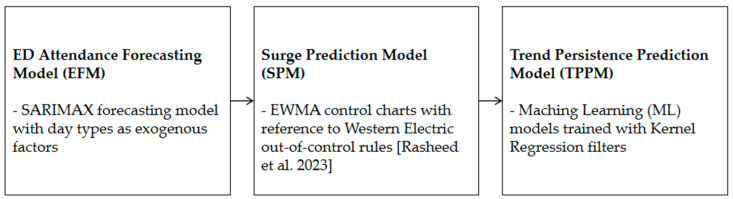
EWS-ED Framework (EFM, SPM, and TPPM) [31].

The structure of the EWS-ED, containing three separate models, is shown in Figure 1. Data from January 2010 to December 2013 were first used to build a SARIMAX model to predict and compare against 2014 data (i.e., four years of training and one year of validation). The prediction errors from 2014 were used to define and initialize an EWMA control chart (the process mean and control limits). This control chart was subsequently used for drift detection for data from January 2015 onwards.

### 2.1. ED Attendance Forecasting Model (EFM)

Univariate analysis was carried out. Time series analysis for ED attendance forecasting was conducted using a SARIMAX model, which is represented as SARIMA(p, d, q) × (P, D, Q)s and is written for a time series $X_{t}$ as [30]: $\phi_{p}\left(B\right)\Phi_{p}\left(B^{s}\right)Y_{t}=\delta +\theta_{q}\left(B\right)\Theta_{Q}\left(B^{s}\right)Z_{t}+\sum_{i=1}^{n}{\beta_{i}w}_{i}$, where B denotes the backward shift operation; $\phi_{p}$, $\Phi_{p}$, $\theta_{q}$, and $\Theta_{Q}$ are polynomials of order p, P, q, and Q, respectively; s is the seasonal period; δ is the drift constant, $Z_{t}\sim WN(0,\sigma^{2})$; $\beta_{i}$ corresponds to the weights of n exogenous factors $w_{i}$; and $Y_{t}=\nabla^{d}{\nabla^{D}}_{s}X_{t}={\left(1-B\right)}^{d}{\left(1-B^{s}\right)}^{D}X_{t}$, where ∇ is the differencing operator, d is the trend difference order, and D is the seasonal differencing order [32]. The exogenous factors describe the types of calendar days. The proposed SARIMAX model was identified by grid search across values of the p, d, q, P, D, and Q parameters that minimized the Akaike Information Criterion (AIC).

Data from 2010 to 2019 were used for training and data from 2020 to 2022 were used for testing. A moving window was used to allocate four years of data for training and one year of data for validation. The accuracy of the retraining framework was evaluated through an incremental learning validation process. During validation, the model parameters were updated with each week of data realization (i.e., incremental learning). Candidate models were diagnostically tested for adequacy using the Ljung–Box Test [33] and Heteroskedasticity Test [34] as well as graphically through a Quantile–Quantile (QQ) Plot and an Autocorrelation (ACF) Plot of the residuals [35]. The underlying assumption of an adequate SARIMAX model is that the residuals will follow a white noise process (i.e., zero mean, constant variance, and uncorrelated) [13,14,15]. The Ljung–Box Test checks for the presence of autocorrelation in the model residuals. It helps to determine whether there are correlation patterns left in the residuals that the model has not captured. The Heteroskedasticity Test assesses whether the variance of the residuals is constant over time. The detection of heteroskedasticity may indicate model misspecification and unreliability of predictive intervals [34]. The QQ Plot compares the distribution of residuals to a normal distribution, helping to visually identify deviations from normality. The ACF Plot shows the autocorrelation of residuals at different lags, providing insight into any remaining patterns that the model has not sufficiently captured.

Model performance was evaluated based on the Mean Absolute Error (MAE), Mean Squared Error (MSE), Mean Absolute Percentage Error (MAPE), and Root Mean Squared Error (RMSE) of annual forecasts. The MAE quantifies the average magnitude of prediction errors, providing a straightforward and interpretable measure of accuracy. The MSE emphasizes larger discrepancies by penalizing larger errors more heavily, making it sensitive to outliers, which is crucial for ED management applications where large errors are undesirable. The MAPE offers a scale-independent relative measure of error, facilitating easy comparison across different datasets. The RMSE is expressed in the same units as the data, and combines the interpretability of MAE with the sensitivity to large errors of MSE. Together, these metrics ensure a balanced assessment of model performance capturing both the average error and the impact of larger deviations [36].

### 2.2. Surge Prediction Model (SPM)

**Figure 2:**
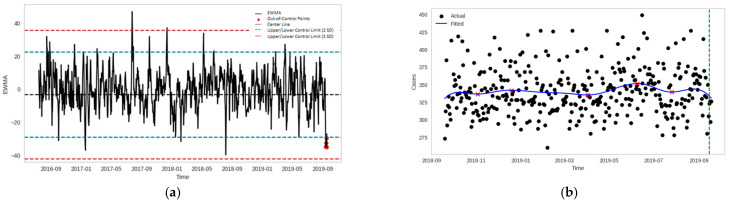
Drift Detection: (**a**) EWMA control chart with OOC condition (SPM) and (**b**) drift signal along the kernel-regressed curve (TPPM filters). The red ‘x’ denotes the local extrema of the kernel-regressed curve.

EWMA control charts serve as an extension to traditional Shewhart control charts [37,38] by providing emphasis on recent data points, and on average have better ability to quickly detect small shifts in the process means compared to the Shewhart Chart [21,39]. EWMA control charts were also shown to be useful for monitoring COVID-19 phases in a recent case study [40]. The upper and lower control limits were set at two standard deviations from the process mean. The EWMA statistics were monitored using resizable windows, for which the size was determined using the Window Resize Algorithm for Batch Data (WRABD) [41]. Initially, past prediction errors were used to establish the upper and lower control limits of the EWMA control chart. As new prediction errors are generated from the weekly streaming data, they can be compared against these control limits. When the prediction errors remain within the control limits, no drift is detected; the errors are stored in a list and accumulate over time. When the majority of prediction errors fall outside the control limits, this signals an out-of-control condition, indicating potential data drift. Therefore, the accumulated errors are added to the existing list of prediction errors and the oldest errors (the tail of the list) are removed in order to maintain a consistent data length. This updated set of errors is used to establish the new control limits. Subsequent prediction errors are monitored, accumulated, and compared against the updated control limits. This iterative process ensures that the control limits evolve in response to data drift. The out-of-control (OOC) rules dictate the sensitivity of the chart to detect drift. The Western Electric rules are often used to detect OOC signals in process control for industrial processes [42]. For a particular week of predictions, we assumed an OOC signal to be detected if at least four out of seven prediction errors (i.e., the majority) were more than two standard deviations from the mean (Figure 2a). The median timestamp of this OOC signal was noted, and is indicated by the dotted green vertical line in Figure 2b.

### 2.3. Trend Persistence Prediction Model (TPPM)

The general trends and turning points in ED attendance were derived through kernel regression [21,39]. A Gaussian kernel regression model was estimated from the ED attendance data and local extrema were noted (Figure 2b). Extrema were defined as the maximum or minimum value within a 30-day neighborhood before and after the point of interest. The bandwidth, which controls the width of the kernel function, was set as 20 to balance the trade-off between new data sensitivity and generalizability.

The OOC condition identified by the EWMA control chart based on the Western Electric rules signals a potential failure of the model to forecast accurately. When drift is detected, the gradients of a kernel regression are computed using local linear regression on various days after the detected drift. These gradients serve as a feature input to a prediction model, with the prediction target being the duration until the next turning point. The gradients from an OOC signal (or drift point) and their corresponding duration to the next turning point serve as predictor–target pairs for the trend persistence prediction model (TPPM). Detected drift points fall into one of two categories: either a drift point near an extremum (i.e., within a 7-day neighborhood vicinity) or a drift point on a trending line. As the interest of the TPPM is to predict the persistence of new trends, drift points in the former context were omitted from the training set.

The number of drift signals (i.e., data points) in the original dataset was insufficient to build a robust TPPM model. Hence, block bootstrapping was performed to resample and increase the training size while preserving the temporal correlations among the attendance data [43,44,45]. Using a block size of 30 (i.e., monthly resampling), seven block bootstrap samples were drawn within each year and concatenated across the years to generate new datasets synthetically.

The drift detection algorithm was applied to each dataset between 2015 and 2019 to generate predictor–target pairs, which were subsequently trained using various prediction models (e.g., Random Forest, XGBoost, Support Vector Machine). The hyperparameters for each potential model were tuned based on a randomized grid search through a 10-fold cross-validation for each hyperparameter candidate set. The performance of each TPPM was tested against the original dataset between 2020 and 2022, then evaluated using the MAE, MSE, RMSE, and MAPE metrics. The bias and variance of the MAE metric were estimated using the jack-knife resampling technique, where the jack-knife estimator was built by aggregating parameter estimators from each subsample obtained by omitting one observation [46]. This approach aims to provide intuition on the stability of model predictions when there are changes in the training set.

## 3. Results

The data comprised 1,700,887 ED attendances spanning a period of 13 years (i.e., 2010–2022). The patients came from diverse community settings, including differing genders, races, residency statuses, and triage classes. The aggregated daily ED attendance was used as the primary source of data. Table 2 summarizes a sample of the study cohort for 2020–2022. Summary statistics for the exogenous variables are listed in Table A1 in Appendix A.

**Figure 3:**
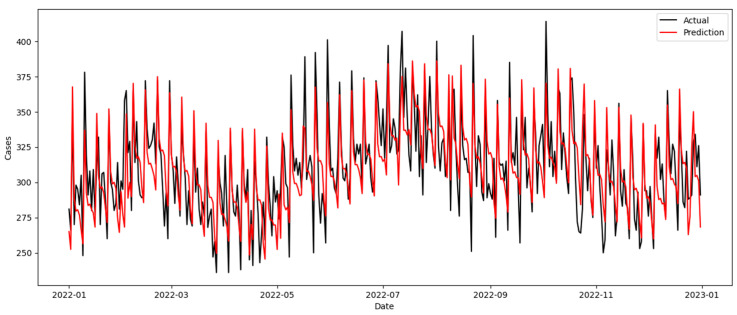
Model forecasts for 2022.

**Figure A1:**
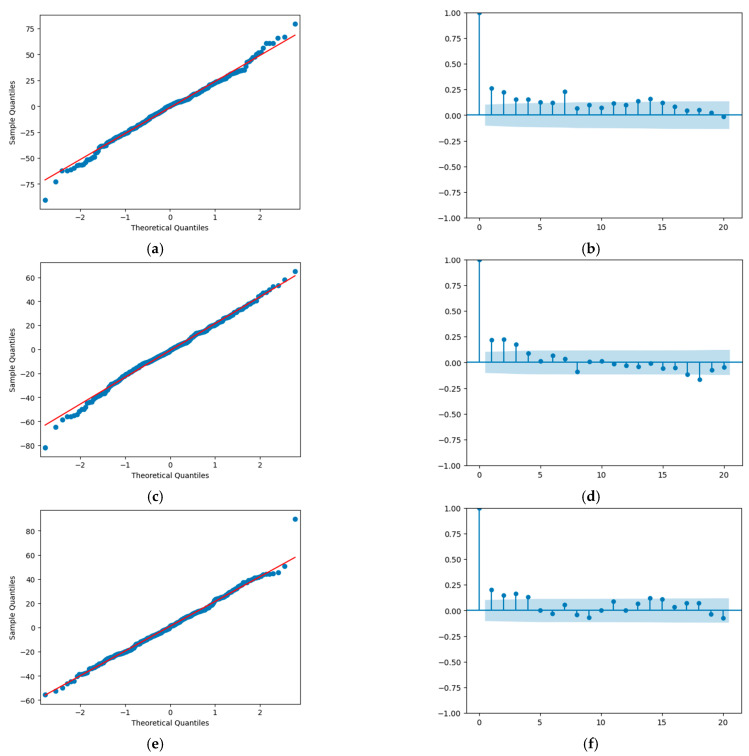
Diagnostic tests: (**a**) Q-Q Plot (2020); (**b**) ACF Plot (2020); (**c**) Q-Q Plot (2021); (**d**) ACF Plot (2021); (**e**) Q-Q Plot (2022); (**f**) ACF Plot (2022). The red line of the Q-Q Plot is a reference line to represent the case where the data is normally distributed. The shaded blue region of the ACF Plot denotes the 95% confidence band to determine if autocorrelation values are statistically significant.

**Figure A2:**
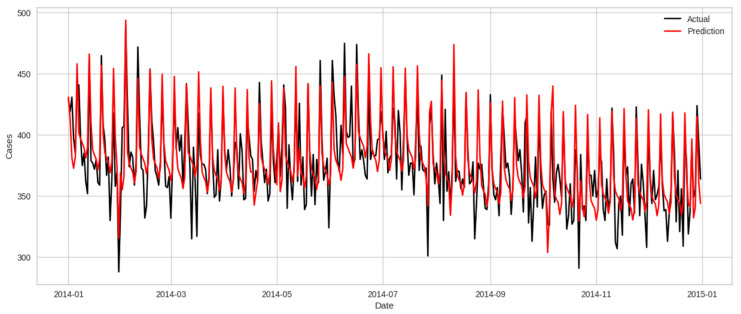
Model forecasts for 2014.

**Figure A3:**
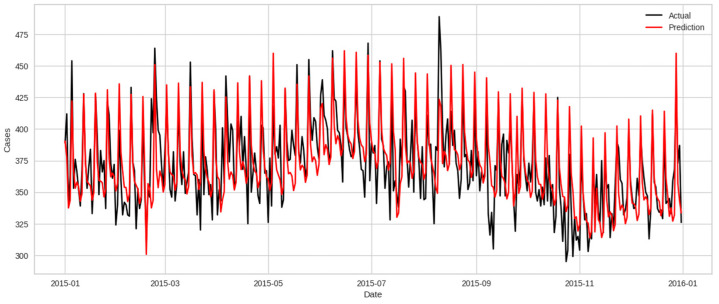
Model forecasts for 2015.

**Figure A4:**
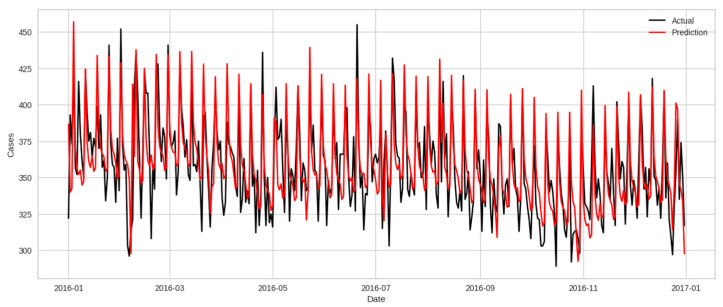
Model forecasts for 2016.

**Figure A5:**
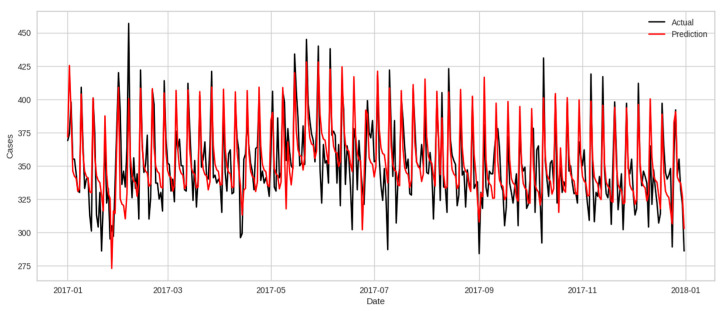
Model forecasts for 2017.

**Figure A6:**
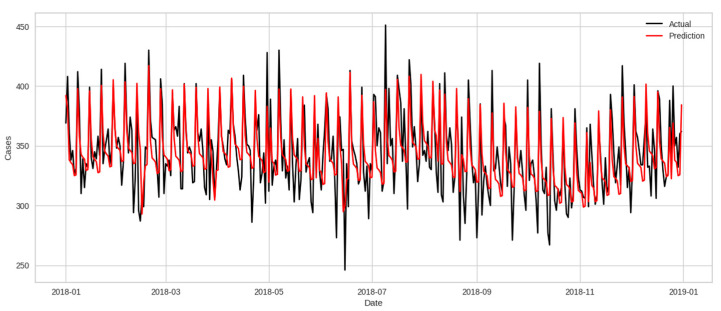
Model forecasts for 2018.

**Figure A7:**
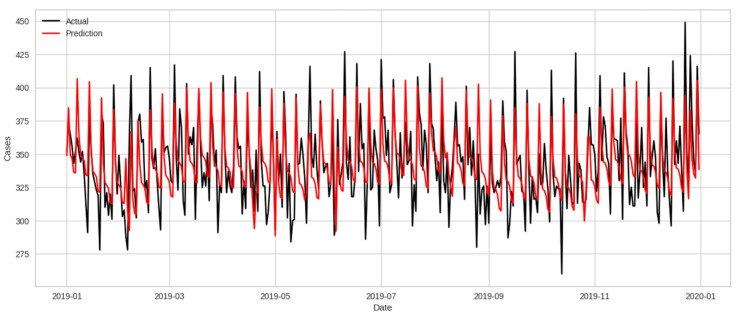
Model forecasts for 2019.

**Figure A8:**
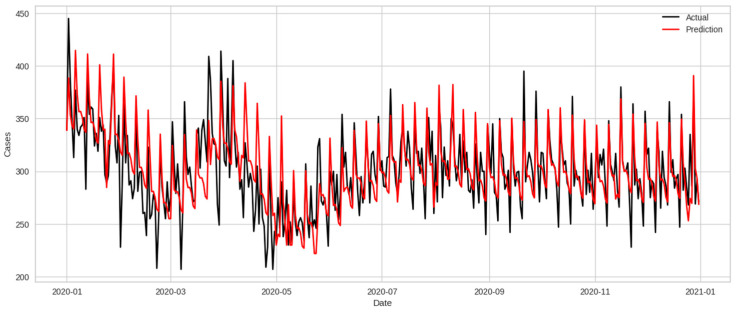
Model forecasts for 2020.

**Figure A9:**
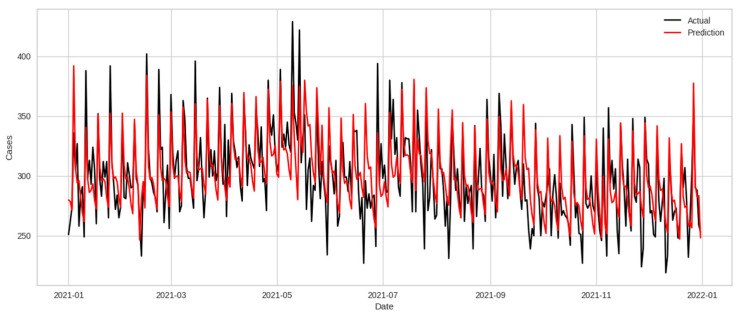
Model forecasts for 2021.

The best-fit models and model performance for the EFM are summarized in Table 3. The validation process converged to two SARIMAX models with parameters (1,1,2)(0,0,0)[7] and (0,1,1)(0,0,0)[7], and the highest error occurred during the test period of 2020. There was no significant evidence of autocorrelation in the errors; all errors were normally distributed, and the variances in the errors were constant across all levels (Table A2 and Figure A1). An example of the time plot of the model forecasts for 2022 is shown in Figure 3. Time plots of the model forecasts for 2014–2021 are shown in Figure A2, Figure A3, Figure A4, Figure A5, Figure A6, Figure A7, Figure A8 and Figure A9. The SARIMAX model allows for the identification of exogenous calendar day-related variables that are significantly associated with ED attendance volumes (Table A3).

**Figure 4:**
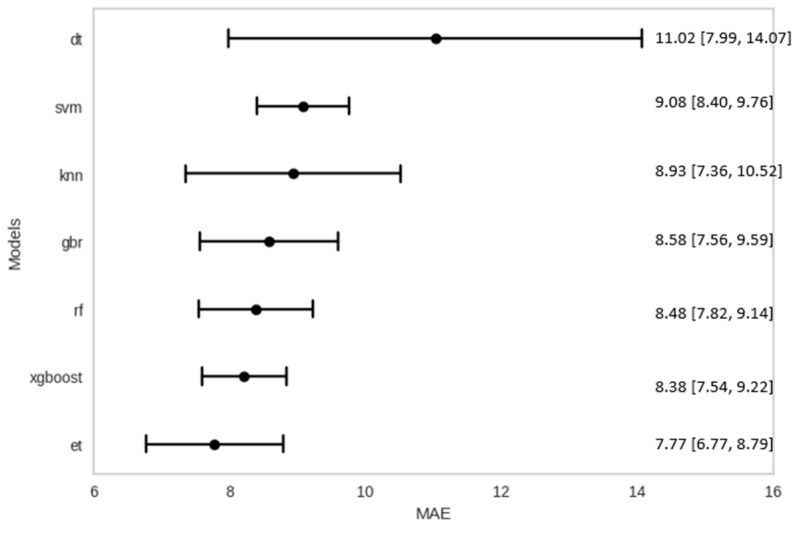
Jack-knife confidence interval for MAE estimates.

**Figure 5:**
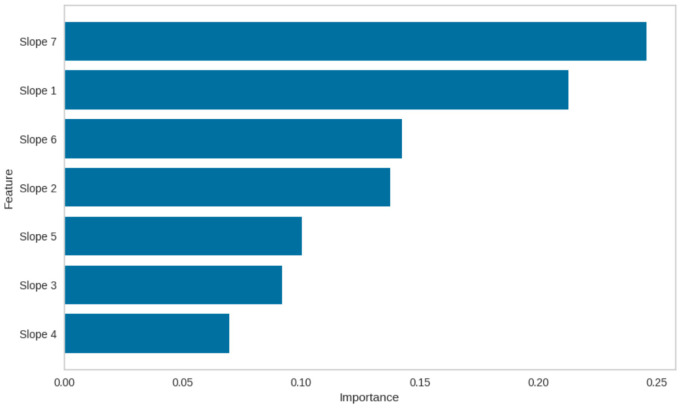
Feature importance for gradients estimated from drift detection within seven days.

Table 4 summarizes the performance metrics of different machine learning models for the TPPM on the test set (i.e., 2020–2022). The bias and variability of the MAE performance were estimated using the jack-knife resampling method; Figure 4 shows the 95% confidence interval (CI) constructed for each MAE estimate, while Figure 5 summarizes the feature importance of the various slopes (or gradients) from kernel regression computed using local linear regression over a seven-day period from the point of drift detection.

## 4. Discussion

This study presents an EWS-ED early warning framework, including a prediction model for the forecasting of ED attendance, a drift detection framework based on process control charts [33], and the ability to predict the persistence of trends after an OOC signal is detected. The time series model enables the identification of independent variables that are significantly associated with ED attendance volumes (Table A3). Compared to the benchmark (i.e., a Sunday that does not fall on a public holiday, post-holiday, or pre-holiday), the ED attendance on post-public holidays was larger on average, with positive and statistically significant magnitudes in the regression coefficients. This concurs with findings reported in the extant literature [13,14]. Similar conclusions can be drawn for Monday, with statistically significant positive associations throughout the test data [47]. The higher ED attendance on these occasions can be attributed to the Monday Effect (i.e., days following a day off) reported in the literature [10]. Possible explanations for the Monday effect include patients returning to the ED from a weekend absence or the return of primary care practitioners to their office and sending their patients to the ED [48]. These analyses support the observation that attendance data falling on post-public holidays and Mondays are essential for accurate ED forecasting.

The time series model in the EFM yielded MAPE values between 5.3% and 6.6%. This result corresponds with the results of similar studies [13]. The time series plot revealed the ability of the SARIMAX model to predict the direction, peak, and troughs consistently and accurately, albeit often at conservative levels (i.e., underestimation) (Figure 3). The model’s worst performance was seen in 2020 (Table 3). The relatively higher error could be due to the COVID-19 outbreak, when EDs worldwide saw a reduction in general attendance [49]; the SARIMAX model may have faced a challenge in addressing the sharp decline in attendance. Similar to findings described in Duarte et al. [50], given the inherent uncertainties resulting from sudden surges or declines in ED attendance volumes due to pandemics or other health emergencies, these results point to the need for a drift detection model to accompany any ED attendance forecasting model [26,27]. Changes in healthcare systems, policies, and other external factors can affect the model’s generalization. The effects of these temporal changes on the ED forecasting framework were accounted for using a time-series-based SARIMAX model for the EFM. From the performance metrics reported in Table A4, the MAE ranged from the smallest in 2017 at 14.6 to the largest in 2015 at 17.2, with an average attendance difference of 2.6, which is relatively small compared to the hundreds of attendances forecasted daily.

**Figure 6:**
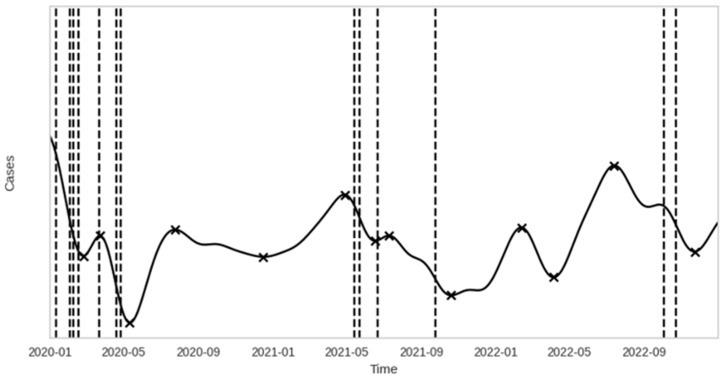
Drift signals between 2020 and 2022 detected by the SPM. The black ‘x’ denotes the local extrema of the kernel-regressed curve.

The drift detection functionality introduced by the SPM leverages the EWMA control chart. EWMA control charts are sensitive to shifts or changes in the distribution of the prediction errors. An OOC signal suggests that the model’s prediction errors exceed the expected variations. This could be due to unusual spikes/dips in ED attendance (e.g., public health crisis, natural disaster), seasonal or temporal shifts in ED attendance (e.g., seasons, holidays), and changes in healthcare policies or resources (e.g., changes in patient flow and capacity management). EWMA and other process control charts have been introduced to detect drifts in the predictive capability of prediction algorithms [51]. By monitoring drifts in the prediction errors, the EWMA chart can provide a simple technique for continuously monitoring predictive accuracy, as the OOC signals provide preemptive information that can identify the potential of sudden and significant changes in data distributions. Figure 6 shows the ability of the EWMA to accurately pick out drift signals where changes in ED attendance are visually apparent.

Although the use of statistical process control methods in the ED setting is not novel [52], the SPM developed on accurate data across the COVID-19 pandemic reveals merits in detecting apparent changes in data trends attributable to events that happened throughout the pandemic in Singapore. A summary of nationwide events that may have led to the corresponding signals picked up by the EWMA chart is shown in Table 5. The period of January 2020 to April 2020 is corroborated with the onset of the COVID-19 pandemic. Health advisories to restrict public movements across workplaces and schools were introduced to curb the rising number of cases [53,54,55,56]. The restrictive measures (i.e., lockdowns, stay-at-home orders, social distancing) reduced the number of patients seeking medical attention at the ED [57]. This reduction may be attributed to decreased non-urgent medical issues, concerns about virus exposure, and increased public awareness about avoiding unnecessary visits to the ED. The drastic declines in ED attendance during the onset of these events were picked up by the EWMA control chart in multiple instances. Nurses and staff at the bed management unit can utilize the signals from the SPM to make preemptive adjustments to the bed resource allocations and staffing requirements.

In 2021 and 2022, Singapore began adapting to the new situation, and drift detections picked up by the EWMA chart occurred at relatively fewer persisting intervals. These signals may represent a temporal shift in ED attendance resulting from holiday effects or external shocks, as compared to changes in ED attendance resulting from the COVID-19 pandemic in early 2020. In these cases, the SARIMAX model alone failed to accurately capture the external effects, leading to higher prediction errors. For example, the drift signal picked up on 23 October 2022 corresponds to the day before the Deepavali public holiday, when the ED often observes reduced attendance. In the same period, local news agencies reported a bed crunch at Singapore hospitals, urging the public to avoid non-emergency visits to the ED [58,59]. The combination of these events led to a notable decrease in ED attendance, complemented by overestimation of ED attendance predictions, causing more significant errors and a corresponding drift signal detected in the EWMA chart. These anomalies indicate the need for a complementary model to inform hospital management of the persistence of trend changes whenever OOC signals are detected.

**Table 5:** Nationwide events.

Detection Date Range	Event Date	Event
8–14 January 2020	2 January 2020	The Ministry of Health (MOH) issued health advisories and temperature checks for passengers at Changi Airport [53]
1–4 February 2020	30 January 2020	Masks are issued to every household to encourage the wearing of masks for those who are unwell [55]
12–18 February 2020	7 February 2020	Disease Outbreak Response System Condition (DOSCORN) level raised from Yellow to Orange [54]
19–24 March 2020	21 March 2020	The first two confirmed deaths due to COVID-19 [60]
15–21 April 2020	3 April 2020	The Circuit Breaker was announced. All non-essential workplaces were closed, and schools were moved to home-based learning [56]
25–28 April 2020		
12–16 May 2021	16 May 2021	Following an uptick in COVID-19 cases, Singapore reverts to stricter restrictions under the name of “Phase 2 (Heightened Alert)” [61]
21–25 May 2021		
22–28 September 2021	27 September 2021	Singapore entered a “Stabilization Phase” [62]
2–6 February 2022	1/2 February 2022	Chinese New Year public holiday, and increasing number of Omicron cases [63]
25 February–1 March 2022	22 February 2022	Singapore hit record-high infection numbers that topped 26,000, signaling the peak and end of the Omicron wave [63]
21–25 October 2022	24 October 2022	Deepavali public holiday. Longer waiting times at hospitals [35,36]

In predicting the extent of trend shifts due to system shocks, the Extra Trees Regressor (ET) model in the TPPM yielded the lowest MAE of 7.54 days, MAPE of 27.4%, MSE of 142.46 days squared, and MAPE of 11.94 days for the TPPM (Table 4). The linear regression (LR) model performed the worst among the tested models. The predictions made by the ET model provided the smallest average MAE of 7.77 days, while the Extreme Gradient Boosting (XGB) model provided the smallest range of 95% CI (Figure 4). The predictive accuracy results of the TPPM points to the fact that pre-emptive planning can be activated if trends are expected to persist for an extended period, helping to deal with ED overcrowding arising from higher ED attendance and bed crunch [64]. Several operational measures can be implemented in the Bed Management Unit (BMU) to ensure optimal resource allocation and patient care. These include increasing staffing levels (i.e., number of doctors and nurses on calls) to manage anticipated rise in patient volume, expanding bed capacity and preparation of non-traditional spaces as additional patient care areas, accelerating discharge planning to facilitate timely bed turnover, and enhancing communication and coordination with other hospitals and care centers to manage patient transfers and support capacity needs effectively [64]. These measures are proposed actions for managing anticipated increases in patient volume. A stricter review of existing policies and thorough evaluation of the efficacy of these measures will be necessary in order to ensure that they effectively address the operational challenges and meet patient care standards.

As part of data preprocessing for the TPPM, drift points detected within a 7-day neighborhood to an extremum (estimated with kernel regression) were omitted in the TPPM training set. This preprocessing aligns with the key aim of the TPPM, which is to predict the persistence of new trends, for which turning points or changes in the direction of trends are not relevant. Nonetheless, further testing was performed on the case where these extreme drift points were included in the training set. Table 6 provides a summary of the test statistics. Compared to the metrics shown in Table 4, deterioration in the performance metrics, for example MAE, is observed for the ET, RF, XGB, SVM, and DT models. This comparison validates the improvement of the proposed data preprocessing procedure to achieve the key objective of the TPPM. The model predictions reveal higher errors for drift points within the vicinity of an extremum (Table A4), which reinforces the fact that trend persistence predictions at extreme drifts can be viewed as false positives. In the operational context (i.e., week-to-week realization of ED attendance) it is not possible to identify whether a set of gradients constitutes a turning point due to the need for future data. As such, a limitation of the algorithm is that predictions are still made for every drift point. Nevertheless, in due time the TPPM could warn users about plausible erroneous predictions when drifts are ascertained as being near an extremum.

Integrating the SPM and the TPPM within the EWS-ED framework takes inspiration from the concept of the drift detection problem [51], extending from pure drift detection to predicting the extent of drift over time. Predicting the extent of drift provides additional information to decision-makers, aiding in the sustainable deployment of ML models and helping to monitor their performance over time [65]. Application of the TPPM for concept drift detection may extend beyond the context of ED attendance. In separate contexts, developers and users can develop models specific to their contexts and utilize the information as a decision support tool to calibrate the smoothing parameter of the EWMA statistic, redefine the OOC conditions, and evaluate appropriate model updating or retraining strategies that suit the use case, among other considerations. This study contributes to the body of knowledge around monitoring concept drift in time series models. It emphasizes the importance of evaluating concept drift from the conflicting problem arising due to the stability versus the plasticity of predictive models over time [66]. Future research will need to quantify the impact of these conflicting objectives in a more theoretical manner.

A limitation of the present study is that the framework has been developed based on a single study site, which may limit the generalizability of results beyond the SH. However, the SH is one of the largest comprehensive public hospitals in Singapore, while the cohort comprised 1,700,887 ED attendances spanning 13 years (i.e., 2010–2022) and appears to be representative of the national population (Table 2). As of 2023, the three largest ethnic groups in Singapore are Chinese, Malay, and Indian, comprising 75.6%, 15.1%, and 7.6% of the total population, respectively [67]. These ratios are consistent with the distribution in the study cohort. The gender distribution of Singaporeans is 97.6 males per 100 females, compared to the study cohort of 104.6 males per 100 females in 2022. The average percentage of Singaporean citizens who attended the hospital emergency department was 86.5% (Table 2), compared to the national proportion of 61.0% [67]. A relatively higher percentage of Singaporean citizens visit public hospitals due to the extensive support and subsidies provided by the government. With the contextual validity of the cohort and the model developed with the SH data, the SH has piloted the EFM module for operational usage since January 2023. Nonetheless, given the consistent public health policies governing emergency care services across Singapore, the modeling framework can potentially be generalized nationally. Future research will look at the external validation of the modeling framework across other public hospitals in Singapore. In order to further validate the model and to ensure its applicability across diverse settings, future research could look into including data from multiple hospitals across different regions to capture a wider range of patient demographics and hospital practices. Briefly, the multi-site study design includes: first, the identification of hospitals across different geographic locations and healthcare systems; second, curation of standardized protocols for data collection, data processing, model implementation and performance evaluation for consistency across participating sites; third, fine-tuning and customization of the EWS-ED framework to account for site-specific variations and unique operational conditions; fourth, analysis comparing model accuracy and effectiveness; and lastly, conducting pilot programs at selected sites to test model feasibility and effectiveness prior to full-scale development. Through a robust multi-site validation approach, this study remains committed to advancing research with enhanced generalizability and ensuring its effectiveness in diverse operational environments [68].

## 5. Conclusions

This research advances emergency healthcare management by introducing a proactive surge detection framework, which is vital for bolstering the preparedness and agility of emergency departments amid unforeseen health crises. The EWS-ED framework is designed to detect drifts and predict the extent of these drifts, thereby allowing pre-emptive signals to be detected in order to consider model updating or retraining. This capability underpins agile operational planning by offering critical signals indicating the need for model recalibration.

## Figures and Tables

**Table 1:** Exogenous factors.

Factor	Description
Holiday	Public holidays declared by the Ministry of Manpower in Singapore (e.g., Good Friday)
Post-Holiday	The working day following a public holiday
Pre-Holiday	The day preceding a public holiday
Working Day	Indicator for weekdays and non-public holidays
Day of the Week	Indicator for Monday, Tuesday, Wednesday, Thursday, Friday, Saturday, or Sunday

**Table 2:** Cohort summary data of daily ED attendance.

Characteristic *	Daily Mean (Standard Deviation)			Daily Median (Interquartile Range)		
	2020	2021	2022	2020	2021	2022
Male	160.8 (23.0)	156.5 (20.8)	160.5 (19.6)	158 (144–231)	154 (143–168)	158.5 (147–172)
Female	141.3 (22.4)	143.3 (19.6)	152.0 (18.9)	140 (125–155)	142 (130–156)	151.5 (138–164)
Chinese	192.2 (28.4)	194.7 (25.9)	204.2 (24.2)	191 (174–209)	192 (178–211)	202 (188–220)
Malay	31.5 (7.74)	31.2 (6.58)	33.5 (7.59)	36 (31–61)	31 (27–35)	33 (28–38)
Indian	42.8 (8.31)	42.6 (8.23)	41.0 (7.39)	48 (42–48)	43 (37–48)	41 (36–46)
Others	15.9 (4.92)	15.8 (3.80)	17.8 (4.63)	19 (15–42)	16 (13–18)	17 (14–21)
Singapore Residents	256.3 (37.0)	261.7 (34.4)	272.9 (33.0)	255 (230–277)	258 (239–281)	271 (250–292)
Non-Residents	45.8 (13.7)	38.2 (7.30)	39.5 (7.47)	43 (36–53)	38 (33–43)	39 (34–45)
P1	30.9 (7.91)	33.5 (7.97)	40.4 (9.44)	31 (25–35.8)	33 (28–39)	39 (34–46)
P1F	0.83 (1.12)	1.09 (1.17)	1.55 (1.67)	0.0 (0.0–1.0)	1.0 (0.0–2.0)	1.0 (0.0–2.0)
P2	83.2 (19.3)	92.6 (18.8)	103.6 (19.9)	82 (70–94)	92 (79–105)	102.5 (90–117)
P2+	85.3 (14.2)	87.8 (13.9)	86.9 (12.4)	85 (76.2–94.0)	87 (79–97)	86 (79–95)
P2F	6.02 (3.33)	7.74 (4.55)	9.02 (6.12)	6.0 (4.0–8.0)	7.0 (4.0–10)	7.0 (5.0–12.3)
P3	56.9 (19.0)	54.8 (11.8)	58.0 (13.1)	54.5 (42–66.8)	54 (47–62)	58 (48–67)
P3F	36.3 (24.5)	21.7 (7.27)	10.4 (9.81)	31 (23–43)	21 (17–27)	7.0 (4.0–14)
P4	0.60 (0.94)	0.40 (0.65)	0.51 (0.75)	0.0 (0.0–1.0)	0.0 (0.0–1.0)	0.0 (0.0–1.0)

* Characteristics are listed in the following order: Gender, Race, Residency Status, Triage Class. Missing data are present under the Race and Triage Class categories: 0.1% and 2.6%, respectively.

**Table 3:** Summary of forecasting performance.

Train Period	Test Period	Best Fit Model	MAE	MAPE	MSE	RMSE
2016–2019	2020	SARIMAX(0,1,1)(0,0,0)[7]	19.5	0.0662	634	25.2
2017–2020	2021	SARIMAX(1,1,2)(0,0,0)[7]	17.4	0.0592	502	22.4
2018–2021	2022	SARIMAX(0,1,1)(0,0,0)[7]	16.3	0.0529	421	20.5

**Table 4:** Summary of TPPM performance.

Model	MAE	MAPE	MSE	RMSE
Support Vector Regression (SVM)	9.08	0.35	144.00	12.00
K Neighbors Regressor (KNN)	8.62	0.30	152.62	12.35
Extra Trees Regressor (ET)	7.54	0.27	142.46	11.94
Random Forest Regressor (RF)	8.38	0.29	154.38	12.43
Extreme Gradient Boosting (XGB)	8.69	0.37	162.54	12.75
Decision Tree Regressor (DT)	9.46	0.34	149.00	12.21
Gradient Boosting Regressor (GBR)	8.85	0.33	180.85	13.45
Linear Regression (LR)	2.72 × 10^7^	1.23	2.32 × 10^15^	4.82 × 10^7^

**Table 6:** Summary of TPPM performance without dropping extreme drift points).

Model	MAE	MAPE	MSE	RMSE
Support Vector Regression (SVM)	7.85	0.31	126.92	11.27
K Neighbors Regressor (KNN)	8.38	0.28	148.23	12.18
Extra Trees Regressor (ET)	9.92	0.46	203.31	14.26
Random Forest Regressor (RF)	12.08	1.96	315.31	17.76
Extreme Gradient Boosting (XGB)	12.08	1.20	319.46	17.87
Decision Tree Regressor (DT)	13.46	2.71	351.31	18.74
Gradient Boosting Regressor (GBR)	8.62	0.45	178.31	13.35
Linear Regression (LR)	3.38 × 10^7^	1.83	3.59 × 10^15^	5.99 × 10^7^

## Data Availability

Data from this research is available via: https://datadryad.org/stash (accessed on 14 June 2023).

## Appendix A

**Table A1:** Summary statistics of exogenous variables.

Factor	Daily Mean (Standard Deviation)			Daily Median (Interquartile Range)		
	2020	2021	2022	2020	2021	2022
Chinese New Year	334.7 (38.0)	264.0 (43.8)	326.5 (44.5)	336.0 (38.0)	264.0 (31.0)	326.5 (31.5)
Christmas Day	268.0 (-)	265.0 (-)	290.0 (1.4)	268.0 (-)	265.0 (-)	290.0 (1.0)
Deepavali	283.0 (-)	246.0 (-)	281.0 (-)	283.0 (-)	246.0 (-)	281.0 (-)
Good Friday	283.0 (-)	266.0 (-)	245.0 (-)	283.0 (-)	266.0 (-)	245.0 (-)
Hari Raya Haji	260.0 (-)	270.0 (-)	350.5 (38.9)	260.0 (-)	270.0 (-)	350.5 (27.5)
Hari Raya Puasa	284.5 (54.4)	330.0 (-)	296.0 (-)	284.5 (38.5)	330.0 (-)	296.0 (-)
Labour Day	238.0 (-)	303.0 (-)	284.0 (14.1)	238.0 (-)	303.0 (-)	284.0 (10.0)
National Day	318.0 (45.3)	278.0 (-)	280.0 (-)	318.0 (32.0)	278.0 (-)	280.0 (-)
New Year’s Day	339.0 (-)	251.0 (-)	281.0 (-)	339.0 (-)	251.0 (-)	281.0 (-)
Vesak Day	282.0 (-)	281.0 (-)	305.5 (13.4)	282.0 (-)	281.0 (-)	305.5 (9.5)
Pre-Chinese New Year	289.0 (-)	260.0 (-)	301.0 (-)	289.0 (-)	260.0 (-)	301.0 (-)
Pre-Christmas Day	285.0 (-)	232.0 (-)	288.0 (-)	285.0 (-)	232.0 (-)	288.0 (-)
Pre-Deepavali	317.0 (-)	254.0 (-)	264.0 (-)	317.0 (-)	254.0 (-)	264.0 (-)
Pre-Good Friday	318.0 (-)	343.0 (-)	309.0 (-)	318.0 (-)	343.0 (-)	309.0 (-)
Pre-Hari Raya Haji	338.0 (-)	377.0 (-)	320.0 (-)	338.0 (-)	377.0 (-)	320.0 (-)
Pre-Hari Raya Puasa	254.0 (-)	344.0 (-)	274.0 (-)	254.0 (-)	344.0 (-)	274.0 (-)
Pre-Labour Day	243.0 (-)	320.0 (-)	286.0 (-)	243.0 (-)	320.0 (-)	286.0 (-)
Pre-National Day	310.0 (-)	231.0 (-)	354.0 (-)	310.0 (-)	231.0 (-)	354.0 (-)
Pre-New Year’s Day	271.0 (-)	253.0 (-)	291.0 (-)	271.0 (-)	253.0 (-)	291.0 (-)
Pre-Vesak Day	252.0 (-)	309.0 (-)	314.0 (-)	252.0 (-)	309.0 (-)	314.0 (-)
Post-Chinese New Year	398.0 (-)	402.0 (-)	365.0 (-)	398.0 (-)	402.0 (-)	365.0 (-)
Post-Christmas Day	372.0 (-)	355.0 (-)	329.0 (-)	372.0 (-)	355.0 (-)	329.0 (-)
Post-Deepavali	380.0 (-)	340.0 (-)	348.0 (-)	380.0 (-)	340.0 (-)	348.0 (-)
Post-Good Friday	327.0 (-)	369.0 (-)	315.0 (-)	327.0 (-)	369.0 (-)	315.0 (-)
Post-Hari Raya Haji	362.0 (-)	355.0 (-)	407.0 (-)	362.0 (-)	355.0 (-)	407.0 (-)
Post-Hari Raya Puasa	331.0 (-)	422.0 (-)	332.0 (-)	331.0 (-)	422.0 (-)	332.0 (-)
Post-Labour Day	290.0 (-)	389.0 (-)	- (-)	290.0 (-)	389.0 (-)	- (-)
Post-National Day	344.0 (-)	348.0 (-)	363.0 (-)	344.0 (-)	348.0 (-)	363.0 (-)
Post-New Year’s Day	445.0 (-)	336.0 (-)	346.0 (-)	445.0 (-)	336.0 (-)	346.0 (-)
Post-Vesak Day	230.0 (-)	312.0 (-)	389.0 (-)	230.0 (-)	312.0 (-)	389.0 (-)
Working Day	310.8 (36.3)	311.0 (34.8)	321.0 (32.1)	307.0 (46.25)	307.0 (46.0)	318.0 (39.0)
Friday	296.6 (31.8)	295.2 (32.0)	305.2 (24.1)	293.0 (37.0)	298.0 (35.0)	305.5 (29.5)
Monday	349.3 (28.6)	352.8 (30.5)	354.6 (31.2)	350.0 (34.0)	353.5 (31.25)	360.0 (38.5)
Saturday	282.7 (32.0)	281.3 (22.9)	296.6 (21.1)	284.0 (31.0)	282.5 (29.25)	293.0 (28.0)
Sunday	268.8 (27.8)	268.5 (25.8)	276.9 (24.4)	266.0 (38.25)	270.0 (39.25)	276.0 (34.25)
Thursday	300.0 (32.6)	295.6 (27.4)	309.4 (30.0)	298.0 (36.0)	293.5 (40.5)	309.0 (36.75)
Tuesday	308.3 (33.2)	304.9 (24.4)	321.4 (27.4)	308.0 (39.0)	305.5 (34.0)	320.0 (31.25)

**Table A2:** Diagnostic tests.

Model (Test Set)	Heteroskedasticity Test		Ljung-Box Test	
	Test Statistic	*p*-Value	Test Statistic	*p*-Value
2020	1.02	0.84	1.71	0.19
2021	1.44	0.00	0.21	0.65
2022	1.02	0.79	0.38	0.54

**Table A3:** Univariate analysis of SARIMAX models.

Testing Period	2020		2021		2022	
Predictors	Coefficient	*p*-Value	Coefficient	*p*-Value	Coefficient	*p*-Value
Intercept	367.4	0.0 *	397.8	0.0 *	429.3	0.0 *
Chinese New Year	5.9	0.4	15.1	0.0 *	8.2	0.2
Christmas Day	−0.1	1.0	−4.1	0.8	−0.5	1.0
Deepavali	−0.8	1.0	−0.7	1.0	−3.6	0.9
Good Friday	−2.4	0.9	−4.7	0.8	−2.7	0.9
Hari Raya Haji	−2.3	0.8	−10.7	0.2	−11.0	0.2
Hari Raya Puasa	−13.7	0.1	−12.3	0.1	−0.9	0.9
Labour Day	−9.8	0.6	−5.4	0.7	−3.7	0.8
National Day	3.3	0.9	0.6	1.0	−7.3	0.6
New Year’s Day	13.3	0.1	14.4	0.4	2.7	0.9
Vesak Day	1.9	0.8	11.3	0.2	10.7	0.3
Pre-Chinese New Year	−49.3	0.0 *	−48.0	0.0 *	−51.5	0.0 *
Pre-Christmas Day	−24.2	0.1	−21.8	0.2	−25.4	0.0 *
Pre-Deepavali	−18.0	0.1	−8.3	0.3	−6.7	0.6
Pre-Good Friday	−19.3	0.0 *	−14.1	0.1	7.1	0.6
Pre-Hari Raya Haji	0.7	1.0	7.4	0.6	10.8	0.5
Pre-Hari Raya Puasa	−31.5	0.0 *	−14.8	0.0 *	−9.7	0.2
Pre-Labour Day	1.8	0.9	4.5	0.7	2.5	0.8
Pre-National Day	−0.3	1.0	4.1	0.7	−7.6	0.4
Pre-New Year’s Day	−24.9	0.0 *	−29.9	0.0 *	−26.5	0.1
Pre-Vesak Day	6.1	0.6	7.3	0.6	−0.3	1.0
Post-Chinese New Year	58.7	0.0 *	59.3	0.0 *	55.8	0.0 *
Post-Christmas Day	46.4	0.0 *	40.1	0.0 *	35.5	0.0 *
Post-Deepavali	18.9	0.0 *	27.6	0.0 *	41.4	0.0 *
Post-Good Friday	−3.6	0.8	−8.9	0.3	−2.2	0.8
Post-Hari Raya Haji	21.4	0.0 *	13.5	0.1	28.6	0.0 *
Post-Hari Raya Puasa	25.1	0.2	32.5	0.1	50.8	0.0 *
Post-Labour Day	39.7	0.0 *	25.9	0.0 *	14.8	0.1
Post-National Day	60.2	0.1	48.3	0.0 *	44.6	0.0 *
Post-New Year’s Day	28.5	0.0 *	47.6	0.0 *	34.7	0.0 *
Post-Vesak Day	27.0	0.0 *	22.9	0.0 *	21.9	0.0 *
Friday	−20.4	0.0 *	−5.8	0.3	−7.2	0.3
Monday	40.1	0.0 *	52.5	0.0 *	48.9	0.0 *
Saturday	3.2	0.1	7.3	0.0 *	9.9	0.0 *
Thursday	−14.5	0.0 *	−1.2	0.8	−3.5	0.6
Tuesday	−1.6	0.8	8.9	0.1	4.5	0.5
Wednesday	−14.8	0.0 *	−3.1	0.6	−4.4	0.5
Working Day	34.3	0.0 *	25.8	0.0 *	31.6	0.0 *
AR1	-	-	1.0	0.0 *	-	-
MA1	−0.9	0.0 *	−1.8	0.0 *	−0.8	0.0 *
MA2	-	-	0.8	0.0 *	-	-

* Statistically significant (*p*-value < 0.05).

**Table A4:** Summary of performance metrics on the validation set.

Train Period	Test Period	MAE	MAPE	MSE	RMSE
2010–2013	2014	16.330	0.044	441.303	21.007
2011–2014	2015	17.207	0.046	489.659	22.128
2012–2015	2016	16.215	0.046	430.955	20.759
2013–2016	2017	14.614	0.042	340.414	18.450
2014–2017	2018	15.626	0.046	406.285	20.157
2015–2018	2019	17.108	0.050	456.608	21.368
